# Evaluating a retrieval-augmented pregnancy chatbot: a comprehensibility–accuracy-readability study of the DIAN AI assistant

**DOI:** 10.3389/frai.2025.1640994

**Published:** 2025-09-22

**Authors:** P. Valan, Pulidindi Venugopal

**Affiliations:** VIT Business School, Vellore Institute of Technology, Vellore, Tamil Nadu, India

**Keywords:** patient education material, health communication, maternal health, chatbot, patient education

## Abstract

**Introduction:**

Patient education materials (PEMs) often exceed common health literacy levels. Retrieval-augmented conversational AI may deliver interactive, evidence-grounded explanations tailored to user needs. We evaluated DIAN, a RAG-enabled pregnancy chatbot grounded in the NHS Pregnancy Book, using a comprehensibility–accuracy–readability (CAR) framework to compare perceptions between women and clinicians across key perinatal domains.

**Methods:**

We conducted a cross-sectional evaluation with standardized prompts and blinded scoring. Participants were 119 women (18–55 years) and 29 clinicians. After brief CAR training and calibration, all evaluators independently rated the same DIAN responses on 4-point Likert scales across postpartum care, pregnancy health and complications, diet and nutrition, and mental and emotional wellbeing. Between-group differences were tested using the Mann–Whitney U test with Bonferroni adjustment across domains per outcome; effect sizes were summarized with *r* = |Z|/√N and Cliff’s delta. Inter-rater reliability was not estimated, given the independent-rater design.

**Results:**

Differences concentrated in postpartum care. Comprehensibility favored women (*U* = 1206.50, *Z* = −2.524, *p* = 0.012; *r* = 0.207; *Δ* = 0.301). Accuracy also favored women (*U* = 1239.00, *Z* = −2.370, *p* = 0.018; *r* = 0.195; *Δ* = 0.282). Readability favored clinicians (*U* = 1181.50, *Z* = −2.639, *p* = 0.008; *r* = 0.217; *Δ* = 0.315). Other domains showed no significant between-group differences after correction. Radar visualizations mirrored these patterns, with women showing larger comprehensibility/accuracy profiles and clinicians showing larger readability profiles in postpartum care.

**Discussion:**

Grounded in an authoritative national guide, DIAN achieved broadly comparable CAR perceptions across groups, with clinically relevant divergence limited to postpartum care. Women perceived higher comprehensibility and accuracy, while clinicians judged language more readable, suggesting a gap between experiential clarity and professional textual ease. Targeted postpartum refinement, lexical simplification, role-tailored summaries, and actionable checklists may align perceptions without compromising fidelity. More broadly, RAG-grounded chatbots can support equitable digital health education when content is vetted, updated, and evaluated with stakeholder-centered metrics. Future work should examine free-form interactions, longitudinal behavioral outcomes, and ethical safeguards (scope-of-use messaging, escalation pathways, and bias audits).

## 1 Introduction

Patient education is fundamental to quality healthcare, yet significant barriers persist in delivering accessible and comprehensible health information to patients and their families. Online search engines are a common source of health information for patients; however, the reliability and accuracy of these digital resources are questionable (Abdullah et al., 2022). This challenge is particularly pronounced with patient education materials (PEMs), which are educational resources designed to inform patients about their disease or illness (Saunders et al., 2018). Research consistently demonstrates that many PEMs are too complex for patients with less than a high school education (Nattam et al., 2023), creating a critical need to enhance the accessibility and usability of high-quality materials for patients and their families (Slatore et al., 2016). To overcome these barriers and ensure patient understanding, innovative technological solutions are needed.

The aim of this research is to evaluate whether an AI-powered conversational agent, equipped with Retrieval-Augmented Generation (RAG), can improve the comprehensibility, accuracy, and readability of patient education content for diverse user groups, specifically women and healthcare professionals in obstetric care. To address the limitations of current PEMs in accuracy and readability, we consider how advances in AI can directly support clearer, more accessible patient communication.

Artificial intelligence (AI) presents unprecedented opportunities to address these patient education challenges. The implementation of AI in healthcare provides detailed technical support through computer intelligence combined with human intervention (Du et al., 2019). However, successful AI implementation in the medical field requires two components: structured data for machine learning and regular training data to enhance system performance (Jiang et al., 2017). The growing nature of data in healthcare leads to AI implementation faster and covers different verticals of healthcare (Ganesh et al., 2022). The inclusion of AI in healthcare has been found to be supportive in communicable or non-communicable disease diagnostics, assessing the risk of mortality and morbidity, predicting and surveilling the outbreak of diseases, and planning and drafting health policies (Schwalbe and Wahl, 2020). While AI offers many benefits across healthcare, its true impact on patient education is realized through more interactive and personalized tools. Within AI applications, conversational AI specifically targets the comprehension and engagement gaps of static PEMs by enabling tailored, interactive explanations.

The word “conversation” may mislead us into understanding conversational AI (ConAI) as an enterprising system with the ability to converse (Elbanna, 2007), but ConAI’s potential to represent human competence, ability to learn and improvise (Gkinko and Elbanna, 2023), and possess a personality trait (Bavaresco et al., 2020) makes it unique among AI models. The inclusion of ConAI will result in focused customization, leading to anthropomorphism (Fotheringham and Wiles, 2023), and creates an opportunity for more engaging and effective patient education experiences (Speirs, 2015). With the help of ConAI, patients can interact with PEMs in real time and receive information in understandable, context-sensitive language. This interactive approach helps bridge knowledge gaps and reduces anxiety around medical care (Akpan et al., 2025). By simplifying complex medical jargon, ConAI makes educational materials more accessible to patients with varying literacy levels (Nasra et al., 2025). Gathering routine patient queries, educating about medications, procedures, and aftercare, and collecting preliminary information before treatments enable clinicians to focus on more complex tasks and improve workflow efficiency (Hong et al., 2022); thus, ConAI becomes a clinical companion. Using ConAI, we can achieve broad reach demands for public health campaigns, such as vaccinations or chronic disease management, and can deliver patient education on a large scale, making high-quality information available to underserved or remote populations (Mondal et al., 2023). Recent innovations in ConAI include “synthetic patients” AI-driven avatars that simulate challenging patient conversations. These tools are now used for training medical students in soft skills such as delivering bad news, managing emotional responses, and addressing health literacy gaps (Chu and Goodell, 2024). However, the effectiveness of conversational agents depends not only on their language abilities and interactivity but also on the accuracy and currency of the information they provide. Yet, despite these advantages, conversational agents can suffer from inaccuracies, outdated content, and inconsistent intent-matching limitations that necessitate explicit grounding in authoritative evidence.

However, to ensure that conversational outputs are not only engaging but also accurate and current, Retrieval-Augmented Generation (RAG) provides a mechanism to ground responses in trustworthy sources.

The RAG addresses these limitations by coupling retrieval of vetted sources with generation, thereby improving factual correctness, currency, and transparency in patient-facing explanations. In some variants, the retriever and generator are trained end-to-end, retrieving evidence passages via dense question–passage similarity to enhance accuracy and interpretability (Lewis et al., 2020; Oche et al., 2025); this grounding is especially valuable for patient education, where conversations must rely on trustworthy, evidence-based sources.

In general, RAG is a method that helps large language models (LLMs) give better answers by first searching for and using information from outside sources, such as documents (Tian et al., 2025). This capability is especially valuable in the context of patient education, where conversations must draw from trustworthy, evidence-based sources to deliver safe and effective information.

In patient education, ensuring information reliability is paramount. RAG models can cite validated, up-to-date medical literature or institutional guidelines in real time, supporting patient safety and regulatory compliance (Gargari and Habibi, 2025). Using RAG to improve the accuracy, reliability, and specificity of clinical responses, especially in knowledge-intensive medical tasks, can be made easy (Shin et al., 2025). “Medicare” and other medical dialog settings will be leveraging RAG (Agrawal1 et al., 2025) for shared medical decision-making (Shi et al., 2023).

Despite promising advances in conversational AI and the integration of retrieval-augmented models, there remains a lack of empirical evidence regarding their effectiveness in improving patient education comprehensibility, accuracy, and readability, especially across different user groups. This research is expected to demonstrate that leveraging advanced conversational AI can enhance the accessibility and clarity of patient education materials, particularly for lay audiences, offer practical insights into tailoring digital health content for distinct user groups using structured evaluation frameworks, and inform future development and deployment strategies for digital patient education solutions across healthcare settings.

## 2 Related works

To contextualize our study, we examined previous research on healthcare chatbots addressing various scenarios, including maternal and reproductive health. Table 1 highlights examples of chatbots developed for different purposes, including fertility awareness (Maeda et al., 2020), gestational diabetes management (Sagstad et al., 2022), maternal health (Nguyen et al., 2024), and perinatal mental health (Chung et al., 2021). Table 2 details their methodologies, showcasing the advancements and study aims addressed. Most existing studies have either focused on technical performance metrics or limited use cases within narrow clinical contexts. Few have systematically compared the perceptions and understanding of both healthcare professionals and lay audiences, gaps that are critical to address as such technologies become increasingly integrated into patient care. Prior work on healthcare chatbots illustrates promise and common limitations, motivating a closer look at how users actually perceive comprehensibility, accuracy, and readability in real use. Building on these considerations, we focus our study on how different user groups perceive the same chatbot content across key obstetric domains.

**Table 1:** Chatbots developed for patient education and health promotion.

Name of chatbot	Fertility chatbot	Dina	Rosie	Dr. Joy
Purpose	Promote fertility awareness and preconception health	Supports pregnant women with GDM by providing guidance	Provides personalized health education for new mothers	Provides obstetric and mental health care support
Target audience	Women aged 20 to 34 years	Pregnant women with gestational diabetes in Norway	Mothers of color, currently pregnant or with infants <6 months	Perinatal women and their partners in South Korea
Platform	Online website	Online and Norwegian digital health platform	Mobile app (iPhone and Android)	Mobile instant messaging app (KakaoTalk)
Development approach	Pre-programmed scripted chatbot	User-centered design with health expert input	Community-driven design over 3 years	Text-mining with input from 11 medical specialists
Core features	Fertility education, RLP counseling, and knowledge improvement	Blood glucose management, diet tips, and physical activity advice	Parenting advice, child development, and health emergency detection	Obstetric and mental health Q&A, symptom checks, CBT tools
Health focus	Fertility and preconception health	Gestational Diabetes Mellitus (GDM)	Maternal and child health	Obstetrics and mental health
Evaluation method	Three-arm randomized controlled trial	Observational study analyzing chatbot logs	Randomized pilot study with treatment and control groups	Usability testing (7-day contextual study)
Key findings	Improved fertility knowledge and behavior intentions, reduced anxiety	Answered 88.51% of questions, mirrored GDM treatment priorities	Reduced postpartum depression, improved health info accessibility	High usability, positive associations with perceived benefits
Limitations	Technical limitations, low comprehension of user inputs	Limited content scope, need for promotion	App crashes, user dissatisfaction with some responses	Limited intent matching, content requires regular updates
Usability features	Simplified educational content, feedback integration	Low-threshold information access, available anytime	Daily push notifications, video tutorials, FAQs	Multi-language support, dialog buttons for guided flow
Personalization level	Moderate (pre-determined scenarios)	Moderate (focus on GDM-related questions)	High (personalized based on user queries)	Moderate to high (rich knowledge base and synonym dictionary)
Accessibility	Accessible via website	Freely available without registration	Accessible on mobile devices	Available via popular instant messenger
Integration with existing systems	Standalone system without broader integration	Integrated with the national digital health platform	Limited integration; standalone app	Integrated within the KakaoTalk platform
Primary outcomes	Increased fertility knowledge, behavior intentions without anxiety increase	Improved access to GDM-related information	Statistically significant reduction in postpartum depression	Enhanced user engagement with medical Q&A and mental health tools

**Table 2:** Previously Used Chatbot Development Methods for Patient Education and Health Promotion.

Name of chatbot	Unnamed fertility chatbot	Dina	Rosie	Dr. Joy
Data used	Pre-designed fertility and preconception health education scripts from trusted sources like the Japan Society of Obstetrics and Gynecology.	Anonymous dialog logs from 610 interactions categorized into themes such as glucose management, diet, and physical activity.	Community feedback through focus groups, listening sessions, and 73,000 expert-vetted passages from trusted medical sources.	3,524 refined Q&A pairs from South Korea’s largest online maternal care community, enhanced with synonyms and neologisms.
Development methodology	Scripted chatbot with predetermined conversational scenarios; content simplified for readability and casual interaction.	User-centered design with input from clinicians, focusing on self-management education aligned with national GDM guidelines.	Community-driven iterative development over 3 years; built a knowledge base with FAQs and push notifications tailored to user needs.	Developed using KakaoTalk’s AI platform with input from 11 medical specialists, focusing on conversational responses and emotional warmth.
Testing methodology	Three-arm randomized controlled trial with 927 women divided into intervention and control groups, evaluating knowledge, behavior intentions, and anxiety.	Observational study analyzing chatbot logs over two time periods, monitoring query categories and fallback rates.	Randomized pilot study with 29 participants, split into chatbot and control groups, evaluating usage, feedback, postpartum depression, and emergency room visits.	7-day contextual usability test with 15 participants providing feedback on daily chatbot interactions, tracked using emojis and qualitative comments.
Results	Improved fertility knowledge and behavior intentions; reduced anxiety; highlighted the need for better user comprehension capabilities.	Answered 88.51% of questions; reflected GDM treatment priorities; recommended better content and wider promotion.	Significant reduction in postpartum depression; high usability but technical issues like app crashes; recommendations for refining responses and app stability.	High usability and positive benefits; appreciated professional content but noted intent matching limitations and need for periodic updates.

We examine comprehensibility–accuracy–readability (CAR) for a RAG-enabled obstetric chatbot among women and doctors across postpartum care, pregnancy health and complications, prenatal preparation and support, diet and nutrition, mental and emotional wellbeing, birth preferences and experiences, and practical preparations for baby.

1. Does the use of a RAG-powered conversational chatbot improve the comprehensibility, accuracy, and readability of patient education materials compared to standard resources, as evaluated by both women (patients) and healthcare professionals?
2. Are there discernible differences in these perceptions (CAR scores) between lay users and clinicians across common patient education domains such as postpartum care, pregnancy health, diet and nutrition, and mental and emotional wellbeing?
3. What are the implications for personalized and effective digital health education for diverse user groups?

## 3 Materials and methods

### 3.1 Participant recruitment and study setting

A multi-modal recruitment strategy was employed to recruit study participants. Healthcare providers at obstetric clinics were approached and provided with study information materials to facilitate recruitment (Rokicki et al., 2025). Recruitment advertisements were placed within participating clinics to maximize visibility among the target population. Potential participant details were collected, and the research team contacted them for further screening and selection.

The inclusion criteria specify that women participants must be 18 years of age or older. Women who were currently pregnant, had experience in pregnancy in the past, or had closely supported someone during pregnancy were considered participants. Irrespective of their educational background, participants were included only if they were able to read and communicate in English, as all study materials and interviews were conducted in English. Women who have back ground in medicine and any connections with healthcare professionals were excluded to ensure perspectives reflected lay experiences.

Healthcare professionals (doctors) were eligible if they were licensed medical doctors (MBBS or equivalent), had at least 2 years of clinical experience in maternal or prenatal healthcare, were currently practicing or had practiced within the past 5 years, were fluent in English, and provided informed consent. Doctors were excluded if they had previous involvement in developing the AI chatbot or any related study.

We then selected two groups of participants to test the DIAN chatbot: 29 healthcare professionals with substantial years of experience and 119 women.

### 3.2 Identification of key concerns through thematic analysis

We gathered queries from research participants to comprehensively understand the recurring questions and concerns encountered by them. During semi-structured interviews, participants were prompted with open-ended questions such as, “What are some common questions or concerns that come up during pregnancy?,” “Are you aware of any patient education materials related to pregnancy?,” and “Have you considered using the Internet to research those questions?” Responses were transcribed and subjected to qualitative thematic analysis. Two researchers independently reviewed the data and performed inductive coding to identify recurring patterns and themes. Through consensus and iterative discussions, responses were systematically organized into major concern areas. Our analysis revealed that 60% of participants were unaware of any patient education materials relating to pregnancy, and 80% reported relying on the Internet as their primary source of information. One participant highlighted, *“I searched information on the health of the mother and baby, the growth of the baby, and the diet that the mother has to follow. First-time mothers, in particular, are often thoroughly confused about whether it is okay to eat certain foods or sleep in specific positions.”* The most common themes identified through this process included postpartum care, pregnancy health and complications, prenatal preparation and support, diet and nutrition, mental and emotional wellbeing, birth preferences and experiences, and practical preparations for baby.

Data collection and thematic analysis were conducted iteratively, with two researchers independently reviewing interview responses and identifying emergent themes. Recruitment and interviews continued until thematic saturation was reached, defined as the point at which no new major themes or substantive concerns emerged from additional participant input. This process ensured that the thematic domains generated fully reflected the range and diversity of participant experiences and concerns.

### 3.3 Formulation and validation of representative questions

Based on these identified themes, we formulated a set of 50 representative questions, designed to reflect the full breadth and diversity of concerns voiced by participants. This preliminary question set was then reviewed and validated by a panel of experienced healthcare professionals (doctors), who evaluated each question for relevance, clarity, and alignment with the underlying themes. Feedback from the doctors was incorporated to refine and finalize the question set. The resulting 50 questions thus represent a balanced and validated sample of commonly encountered pregnancy-related concerns, which were subsequently used for evaluating chatbot performance with both participant groups.

### 3.4 Adaptation of NHS pregnancy book content for chatbot responses

For the development of the chatbot’s knowledge base and responses, we adopted the entire content from the NHS guidebook, *The Pregnancy Book: Your Complete Guide to a Healthy Pregnancy, Labour and Childbirth, and the First Weeks with Your New Baby*. This comprehensive, evidence-based guide was selected for its breadth, clinical reliability, and national standard status in patient education. The full text was integrated into the chatbot’s responses, ensuring that users had access to holistic and authoritative information on pregnancy-related topics.

During adaptation for chatbot use, language from the original guide was simplified where necessary to accommodate the reading levels of our target population, specifically individuals with less than a high school education. Simplification involved paraphrasing technical terms and complex sentences to enhance understanding, while care was taken to preserve the accuracy and intent of the medical advice. All adapted content underwent review by healthcare professionals to ensure fidelity to the original guidance and suitability for lay users before deployment within the chatbot platform.

### 3.5 Operationalization and assessment of comprehensibility–accuracy–readability (CAR) scores

We adopted the evaluation process detailed in a recent study on developing AI-generated medical responses for cancer patients (Lee et al., 2024), which assessed responses based on the comprehensibility–accuracy–and readability (CAR) scores.

Comprehensibility was defined as the degree to which a response could be easily understood by a lay audience, emphasizing logical flow, coherence, and absence of ambiguity; it was operationalized using PEMAT Understandability criteria (Shoemaker et al., 2014). Accuracy referred to the factual and clinical correctness of the information, its alignment with current obstetric guidelines, and its relevance to the question; it was operationalized by comparing each response against authoritative sources (e.g., the NHS guidelines) and assigning a rating for correctness and completeness (Shepperd and Charnock, 2002). Readability reflected whether language, vocabulary, and sentence structure were suitable for individuals with less than a high school education, avoiding technical jargon and unnecessary complexity; it was operationalized via Flesch–Kincaid Grade Level, targeting a score ≤ 8th-grade level for all responses (Kruse et al., 2020). All responses were rated independently by evaluators using a standardized 4-point Likert scale: 1 = Insufficient, 2 = Moderate, 3 = Good, 4 = Very Good.

Readability of chatbot responses was evaluated using both quantitative and user-centered approaches. For each response, we checked sentence length and structure to ensure the text was simple, concise, and suitable for non-specialist readers. In addition, participants were asked to rate the ease of reading and list any words or phrases they found difficult or unfamiliar. This method follows established patient information evaluation frameworks, which recommend combining simple linguistic analysis with direct feedback from users to improve accessibility and identify barriers to understanding (Garner et al., 2011).

To control for variations in interpretation and engagement, all participants evaluated chatbot responses to the same set of pre-defined questions, rather than chatting freely. Before the evaluation, all evaluators, both doctors and women, were taught how to use the CAR framework. This training included going over sample chatbot answers together and discussing how each of the three CAR categories should be scored. The goal was to make sure everyone understood the criteria in the same way. After initial training, evaluators scored some example responses. If they gave different scores for the same example, the team discussed why. Those discussions helped clarify any ambiguities and align everyone’s judgments. When evaluators scored the real chatbot responses, each answer was stripped of any information identifying who authored it or under what circumstances it was generated. This blinding prevents any conscious or unconscious bias from affecting their scores.

### 3.6 Statistical analysis and visualization

Following data collection, we conducted Mann–Whitney *U* tests (Ruxton, 2006) to compare the responses of doctors and women on CAR across four content areas: (1) postpartum care, (2) pregnancy health and complications, (3) diet and nutrition, and (4) mental and emotional wellbeing. This non-parametric test was chosen because of its suitability for ordinal data and its robustness when dealing with non-normal distributions or unequal group sizes.

**Figure 1:**
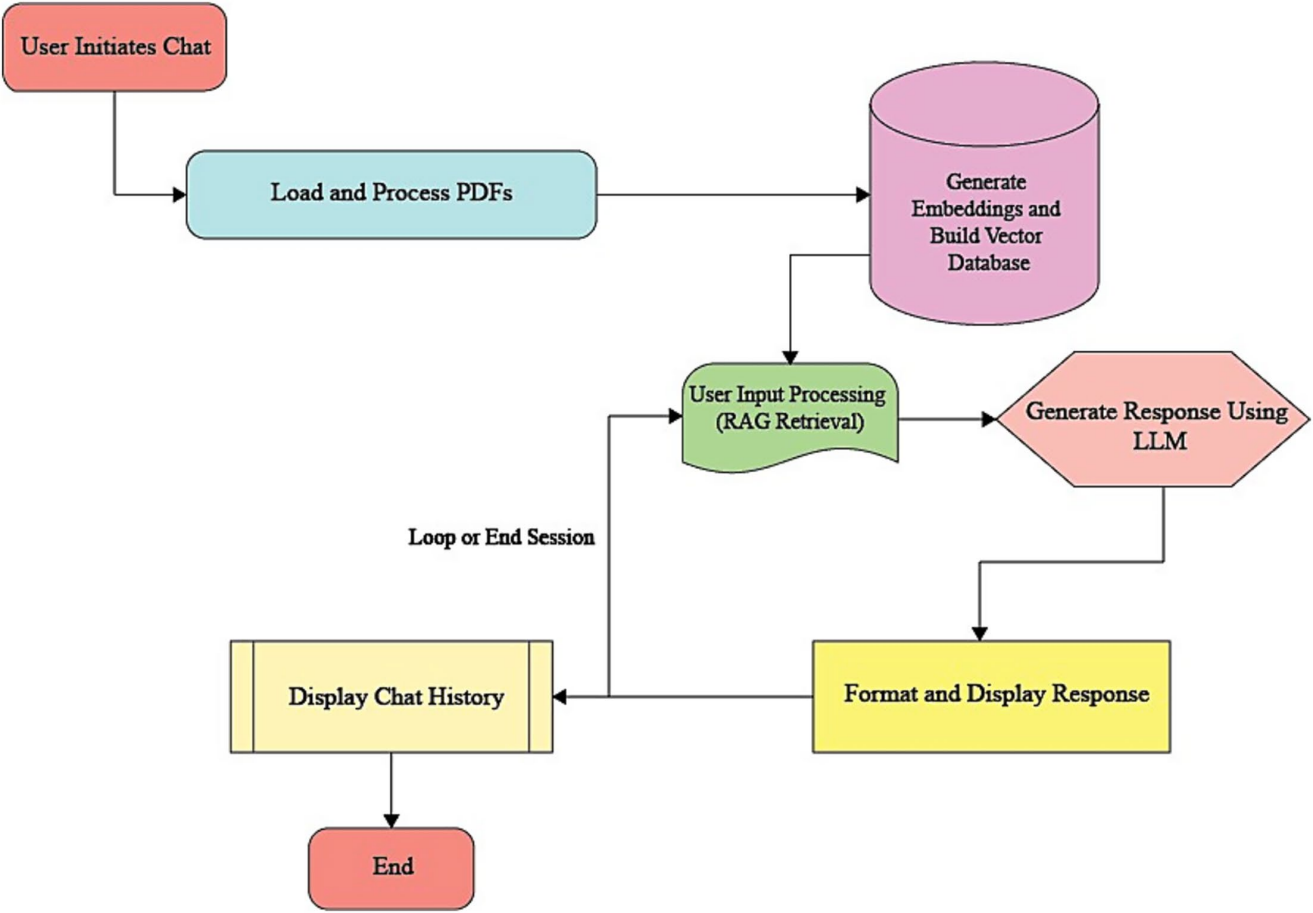
DIAN app architecture.

Between-group comparisons for each rating domain (comprehensibility, accuracy, and readability) were performed using the Mann–Whitney *U* test, appropriate for ordinal 4-point Likert ratings and non-normal distributions. For each comparison, we report the standardized effect size *r* = |*Z*|/√*N* (*N* = *n*1 + *n*2) (Ialongo, 2016), and to complement r, we additionally report Cliff’s delta (*Δ*) to express dominance (stochastic superiority) between groups (Rahlfs and Zimmermann, 2019). Exact/asymptotic *p*-values and tie handling followed the software defaults, and Bonferroni correction was applied across the four domains per outcome. Inter-rater reliability indices were not computed for participant ratings, as respondents functioned as independent raters providing their perceptions, rather than interchangeable judges of the same items for agreement. Thus, our analytic focus was on comparing group rating distributions rather than assessing inter-rater reliability (Figure 1).

**Figure 2:**
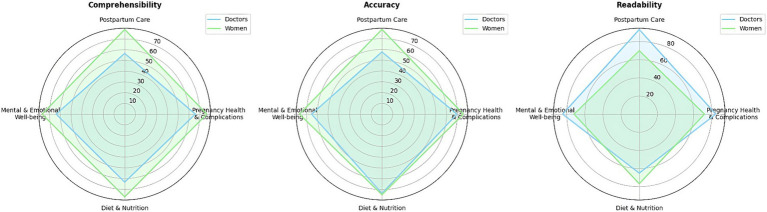
Radar chart comparing doctors’ and women’s ratings.

By leveraging Google Colab, we combined non-parametric statistical analysis with radar charts to graphically present our findings (Figure 2). Radar charts overlay multiple quantitative dimensions in a single, coherent shape, enabling immediate visual detection of systematic differences between groups and across variables. This multidimensional “polygon” format supports holistic comparison without requiring several separate graphs (Schlee et al., 2017). By mapping each content area axis, postpartum care, pregnancy health and complications, diet and nutrition, mental and emotional wellbeing, and plotting mean-rank CAR scores for doctors and women as overlaid polygons, radar plots reveal each group’s distinct strengths and weaknesses in a single view. The relative compactness or elongation of polygons across axes visually encodes dimensional uniformity versus variability (Mason et al., 2024). Radar plots emphasize axes where group polygons diverge most, directing readers to specific content areas that may need targeted improvement (for instance, areas where doctors rate comprehensibility lower than women). This intuitive, at-a-glance interpretation enhances readability for both methodological and clinical audiences. To facilitate interpretation of group comparisons across multiple response domains (comprehensibility, accuracy, and readability), radar charts were constructed for each study group (doctors and women). For each chart, the mean CAR scores for postpartum care, pregnancy health and complications, diet and nutrition, and mental and emotional wellbeing were plotted on separate axes radiating from a common center. This allowed simultaneous visualization of performance across all domains for each group, highlighting strengths and weaknesses in chatbot responses. Radar charts support intuitive comparisons by visually expressing where profiles overlap, diverge, or show relative advantage, thus complementing the statistical tables.

### 3.7 Informed consent

Informed consent was obtained from all participants, both women and healthcare professionals, before their involvement in the study. Participants received detailed information about the study’s purpose, procedures, potential risks, and benefits. Participation was entirely voluntary, and individuals could withdraw at any time without consequence. Confidentiality and anonymity were assured for all responses and data collected.

## 4 Results

This research consisted of two study groups comprising 119 women and 29 doctors. The female study group majority aged 18–25 years (52.1%), followed by 26–40 years (42.0%) and 41–55 years (5.9%). The majority of patient participants had completed school (53.8%), with additional representation from undergraduate (29.4%) and postgraduate (16.8%) educational levels. The doctor group consisted of 21 women and 8 men, aged 26–40 years (65.5%) or 41–55 years (34.5%). The sample sizes (*n* = 29 clinicians; *n* = 119 women) reflect recruitment feasibility during the study window. *Post-hoc* power estimates based on observed effects indicated achieved power of ~0.48–0.57 for the largest contrasts (postpartum care), suggesting the study may be underpowered; we therefore advise larger, balanced samples in future studies. The majority of doctors held postgraduate degrees or higher qualifications. All participants were recruited from a region where English is not the primary language, representing predominantly non-native English speakers. Participant characteristics are detailed in Table 3.

**Table 3:** Demographic characteristics of study participants.

Study group	Gender (F/M)	Age 18–25	Age 26–40	Age 41–55	School	UG	PG	PhD/MD	*N*
Women	119/0	62	50	7	64	35	20	0	119
Doctors	21/8	0	19	10	–	–	18	1	29

Doctors’ and women’s ratings on three important dimensions, readability, accuracy, and comprehension across four content areas (1) postpartum care, (2) pregnancy health and complications, (3) diet and nutrition, and (4) mental and emotional wellbeing were compared using a series of Mann–Whitney *U* tests. Table 4 (comprehensibility), Table 5 (accuracy), and Table 6 (readability) display the findings.

**Table 4:** Comprehensibility.

Group questions (factors)	Mean rank		Mann–Whitney *U* test	*Z*-value	*P*-value	p_Bonf	*r*	Effect size magnitude	Cliff’s Δ	Direction
	Doctor	Women								
Postpartum care	56.60	78.86	1206.500	−2.524	0.012	0.144	0.207	Small–moderate	0.301	Patients > doctors
Pregnancy health and complications	65.86	76.61	1475.000	−1.219	0.223	1	0.1	Small	0.145	Patients > doctors
Diet and nutrition	63.33	77.08	1401.500	−1.575	0.115	1	0.129	Small	0.188	Patients > doctors
Mental and emotional wellbeing	63.93	77.08	1419.000	−1.485	0.138	1	0.122	Small	0.178	Patients > doctors

**Table 5:** Accuracy.

Group questions (factors)	Mean rank		Mann–Whitney *U* test	*Z*-value	*P*-value	p_Bonf	*r*	Effect size magnitude	Cliff’s Δ	Direction
	Doctor	Women								
Postpartum care	57.72	78.59	1239.000	−2.370	0.018	0.216	0.195	Small	0.282	Patients > doctors
Pregnancy health and complications	69.66	75.68	1585.000	−0.680	0.497	1	0.056	Negligible	0.081	Patients > doctors
Diet and nutrition	73.50	74.74	1696.500	−0.141	0.888	1	0.012	Negligible	0.017	Patients > doctors
Mental and emotional wellbeing	65.41	76.71	1462.000	−1.274	0.203	1	0.105	Small	0.153	Patients > doctors

**Table 6:** Readability.

Group questions (factors)	Mean rank		Mann–Whitney *U* test	*Z*-value	*P*-value	p_Bonf	*r*	Effect size magnitude	Cliff’s Δ	Direction
	Doctor	Women								
Postpartum care	93.26	69.93	1181.500	−2.639	0.008	0.096	0.217	Small–moderate	0.315	Doctors > patients
Pregnancy health and complications	84.53	72.05	1434.500	−1.416	0.157	1	0.116	Small	0.169	Doctors > patients
Diet and nutrition	65.09	76.79	1452.500	−1.338	0.181	1	0.11	Small	0.158	Patients > doctors
Mental and emotional wellbeing	84.60	72.04	1432.500	−1.422	0.155	1	0.117	Small	0.17	Doctors > patients

According to Table 4, the only area where there was a statistically significant difference in the comprehension of women and doctors was postpartum care (*U* = 1206.50, *Z* = −2.524, *p* = 0.012). Compared to doctors (mean rank = 56.60), doctors found postpartum care information easier to understand (mean rank = 78.86). To supplement the statistical significance, the effect size for comprehensibility in postpartum care was *r* = 0.207 (small to moderate) and Cliff’s delta = 0.301, indicating that the practical difference between women and doctors was small to moderate. Interestingly, no significant differences were found for the other three content areas: mental and emotional wellbeing, diet and nutrition, and pregnancy health and complications. This suggests that both groups generally had similar opinions regarding the simplicity or ease of understanding the content in these areas.

Since comprehensibility gauges how easily information can be understood, women’s higher scores on postpartum care may reflect their familiarity and experience with the topic, while physicians may evaluate comprehensibility in comparison with a clinical standard. This distinction emphasizes the importance of conveying postpartum care information in a manner that is both clinically accurate and understandable to all audiences, including non-professionals.

The accuracy results (Table 5) also show a significant difference only for postpartum care (*U* = 1239.00, *Z* = −2.370, *p* = 0.018), where women had higher mean ranks (78.59) than physicians (57.72). The effect size for accuracy in postpartum care was *r* = 0.195 (small) and Cliff’s delta = 0.282, suggesting the practical difference was small. Diet and nutrition, mental and emotional wellbeing, and pregnancy health and complications did not show any statistically significant differences.

Accuracy refers to whether the content is factually correct, reliable, and in line with current medical or experiential knowledge. Because of their close connection to and involvement in postpartum events, women may perceive postpartum treatment to be more accurate. In contrast, physicians who use a more rigorous clinical lens might examine the same material more closely. These results suggest a potential discrepancy between the evaluation of factual correctness by professional and lay audiences, underscoring the significance of sophisticated evidence-based communication techniques in postpartum care.

According to Table 6, there is one more significant difference in reading between women and doctors in postpartum care (*U* = 1181.50, *Z* = −2.639, *p* = 0.008). In contrast to women (mean rank = 69.93), doctors notably thought the postpartum care language was easier to understand (mean rank = 93.26). No discernible variations in readability scores were observed across other subject areas. The corresponding effect size for readability in postpartum care was *r* = 0.217 (small to moderate) and Cliff’s delta = 0.315, indicating the practical magnitude of differences was small to moderate.

Readability measures the ease of reading and processing a text. The use of clinical terminology or structure that is more in line with a medical professional’s reading expectations may be the reason for the higher ratings given by physicians for postpartum care. In contrast, women, who might choose simpler, informal language, scored worse on reading tests. This disparity highlights the importance of adjusting presentation style and text complexity to the target audience, especially when discussing delicate subjects such as postpartum care.

Across all assessed domains, reported effect sizes were predominantly small, indicating that while some group differences reached statistical significance, their magnitude was limited in practical terms. This underscores the value of reporting effect sizes alongside *p*-values.

Radar charts provided an immediate, holistic visualization of performance differences between doctors and women across all evaluated domains: comprehensibility, accuracy, and readability. By mapping mean scores for each content area (postpartum care, pregnancy health and complications, diet and nutrition, and mental and emotional wellbeing) onto separate axes and overlaying group polygons, the charts allowed simultaneous comparison of group profiles in a single, intuitive view.

This format made dimensional strengths and weaknesses visibly apparent: For example, the marked expansion of the women’s polygon on the postpartum care axis for comprehensibility and accuracy highlighted their higher ratings in this domain, while the pronounced extension of the doctors’ polygon in readability for the same axis showcased their relative advantage there. The visual contrast between polygons directly echoed patterns detected in the statistical analysis, emphasizing where divergences were most substantial and supporting quick identification of domains needing further improvement.

## 5 Discussion

We examined the performance of the DIAN chatbot in advising women. As we implemented the grading technique based on prior studies, our chatbot promises to be the best integration method while increasing patient education. Postpartum care was the only area where the ratings of doctors and women varied significantly across all three criteria. Women rated postpartum care information higher for accuracy and comprehensibility, indicating that content tailored to postpartum experiences may be better suited to their unique needs and opinions. Postpartum care is not just informational but deeply emotional, with women reporting explicit desire for more emotionally attuned, patient-centered, and understandable support (Roberts et al., 2025). In our study, divergence in postpartum care response within CAR metrics highlights that deeply emotional and precisely tailored communication can have a significant impact. Postpartum-related issues present unique clinical complexities (Fox et al., 2018). Both women and clinicians have distinct expectations and needs in postpartum dialogs, and harder to standardize, especially for AI chatbots. Postpartum content often addresses issues of trauma, depression, and abuse, making it not only technically complex but also deeply emotionally sensitive (Islam et al., 2020). Even small failures in clarity, trust, or appropriateness are amplified because postpartum mothers are acutely attuned to the tone, nuance, and completeness of medical advice (Amar and Sejfović, 2023). The emotionally charged, multidimensional nature of postpartum care, requiring both technical information and emotional reassurance, logically leads to gaps in CAR metrics, with women participants accepting simple content while doctors expect clinical information from the same postpartum material.

Particularly for first-time mothers, the uneven quality or questionable sources of Internet information regarding pregnancy and Childcare can be frightening (Chua et al., 2023). Our findings indicate that postpartum care is a crucial topic with notable variations in readability, correctness, and comprehensibility between expectant women and physicians. These variations imply that different audiences may have different perceptions or interpretations of postpartum content. A previous study with Rosie Chatbot (Nguyen et al., 2024) emphasizes the value of accurate and culturally sensitive postpartum information by using approved, on-demand content. Our research supports the basic idea that Rosie’s design is a useful digital tool for treating postpartum depression. Our work underlines the value of user-centered design and iterative feedback in creating a text-based conversational agent (Calvo et al., 2023). This approach aligns with our method of determining whether various end users find the chatbot content comprehensible and trustworthy. Together, these findings reinforce the importance of designing conversational agents that adapt dynamically to user feedback, employ brief and comprehensible messaging, and remain tightly aligned with clinical best practices.

The findings from our chatbot evaluations spanning postpartum care, pregnancy health and complications, diet and nutrition, and mental and emotional wellbeing mirror key themes from the recent study by Kaphingst et al. (2024), which demonstrated that automated conversational agents can be equivalent to standard of care (SOC) approaches in delivering key health information. In their study, a chatbot guided patients through cancer genetic services, achieving completion rates for pretest counseling and test uptake like those seen with in-person appointments. This equivalence is highly relevant to our context. Although their focus was on cancer risk assessment, the takeaway is that a well-designed chatbot can successfully convey complex medical content, such as postpartum guidelines or nutrition education, to a broad patient audience.

The review of dementia-focused chatbots highlighted limitations in achieving natural, adaptive dialog and comprehensive content delivery (Ruggiano et al., 2021). Their findings imply that many existing systems struggle to balance technical accuracy and user-friendly communication. Our results reinforce this notion that optimizing comprehensibility and accuracy for lay audiences while maintaining readability for expert users is crucial. Both studies underscore that successful chatbot interventions must harmonize evidence-based, accessible content with adaptable conversation flows to meet users’ diverse needs.

Our chatbot study and DR-COVID (Yang et al., 2023) work underscores the promise of AI-driven conversational agents in health education. Our study demonstrates that chatbot responses achieve high comprehensibility and accuracy for lay users, whereas clinicians value technical and scientific readability. Similarly, DR-COVID’s ensemble NLP approach achieved robust overall accuracy (0.838) and top-3 accuracy (0.922) in delivering COVID-19 information across multiple languages. Despite their differing domains, both studies highlight that adaptive, evidence-based chatbot systems can effectively translate complex medical information into user-friendly, reliable guidance.

Our findings highlight the importance of modifying health information for various audiences. For women in the lay audience, accuracy and comprehensibility can be improved by using clear language and practical relevance. Clinical Audience (Doctors) Professional terminology or structure can enhance reading. There were no notable differences between the two groups in terms of diet and nutrition, mental and emotional wellbeing, or pregnancy health and complications. These results imply that there might be a more general agreement or useful resources available for these subjects outside of postpartum care. Ongoing improvement, however, might make use of methods such as audience segmentation, natural language processing, or dynamic text production to better tailor information delivery to the unique requirements of each subgroup in future studies.

## 6 Conclusion

Chatbots are not human clinicians (Manole et al., 2024). They cannot fully replicate a human provider’s ability to interpret complex clinical situations or personalize advice beyond the knowledge base they are trained on (Frodl et al., 2024). Because chatbots rely on existing data sources (Chow et al., 2025), inaccuracies or biases (Van Poucke, 2024) can be perpetuated unless those sources are carefully vetted. Using patient education content, this study adds to the expanding corpus of information on chatbot technology. Research must continue as chatbot technology develops quickly to stay updated with new trends, user behavior, and societal ramifications (Følstad et al., 2021). Chatbots offer a quick, easy, and varied way to exchange information (Yu et al., 2023). It is crucial to employ chatbots appropriately, solve ethical issues, and carry out additional research to fully reap the benefits of this technology in medical and health sciences (Razak et al., 2023).

## Data Availability

The raw data supporting the conclusions of this article will be made available by the authors, without undue reservation.
